# Supplementary figures and images for: A New Strategy for Somatotype Assessment Using Bioimpedance Analysis: Stratification According to Sex
Source: J Funct Morphol Kinesiol. 2022 Oct 14;7(4):86. doi: 10.3390/jfmk7040086 (PMC9590076; doi:10.3390/jfmk7040086)

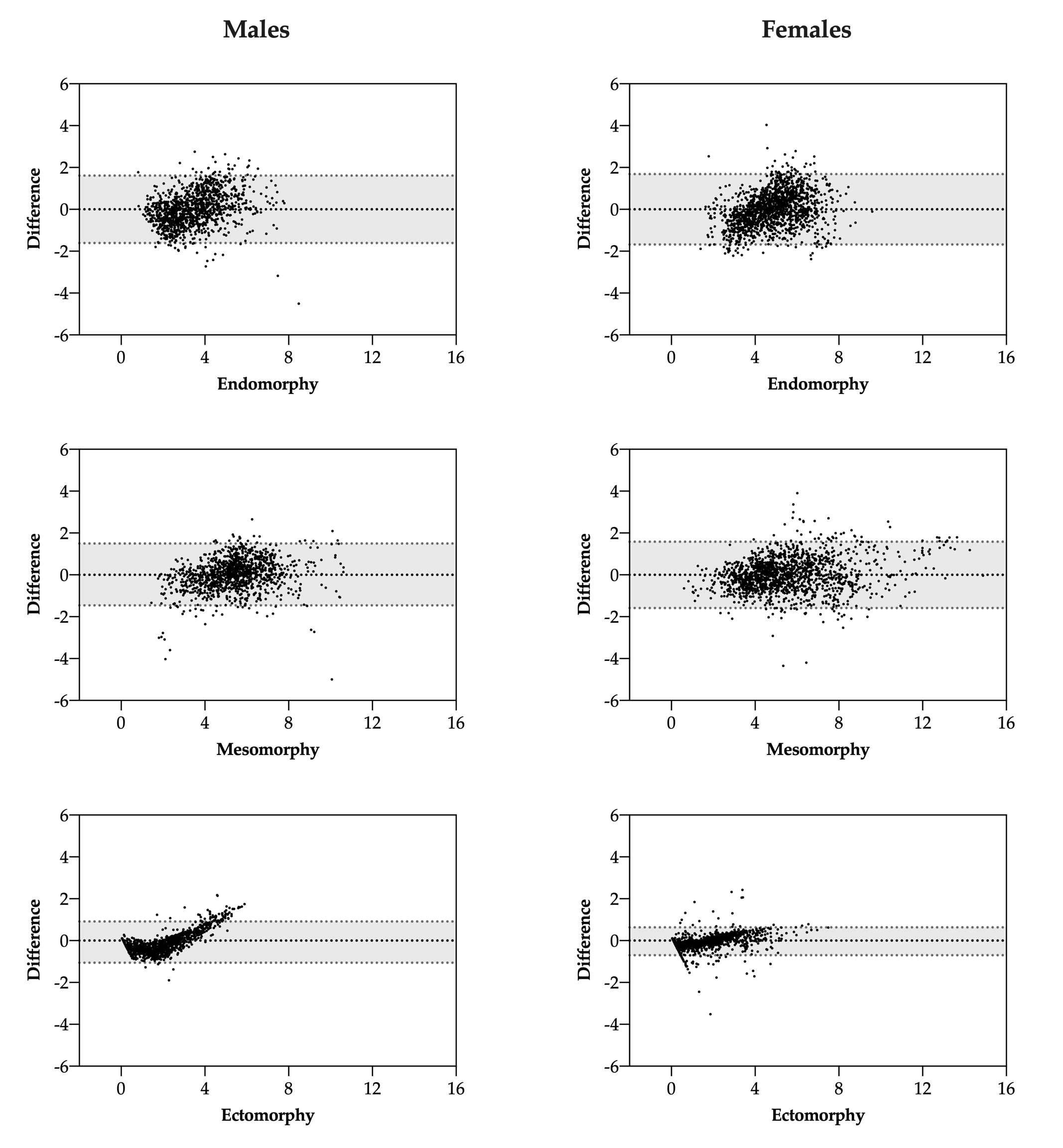

Supplement: Supplementary file 1 [file jfmk-07-00086-s001.zip › jfmk-1943500-supplementary.tiff]
